# The Genetic Population Structure of Robinson Crusoe Island, Chile

**DOI:** 10.3389/fgene.2020.00669

**Published:** 2020-06-26

**Authors:** Hayley S. Mountford, Pía Villanueva, María Angélica Fernández, Lilian Jara, Zulema De Barbieri, Luis G. Carvajal-Carmona, Jean-Baptiste Cazier, Dianne F. Newbury

**Affiliations:** ^1^Department of Biological and Medical Sciences, Faculty of Health and Life Sciences, Oxford Brookes University, Oxford, United Kingdom; ^2^Department of Speech Language and Hearing Sciences, Faculty of Medicine, University of Chile, Santiago, Chile; ^3^Human Genetics Division, Faculty of Medicine, Institute of Biomedical Sciences, University of Chile, Santiago, Chile; ^4^Directorate of Academic Development, Academic Vice-Rectory, Research and Postgraduate, Saint Thomas University, Santiago, Chile; ^5^Department of Biochemistry and Molecular Medicine, School of Medicine Genome Center, University of California, Davis, Davis, CA, United States; ^6^Centre for Computational Biology, University of Birmingham, Birmingham, United Kingdom; ^7^Institute of Cancer and Genomic Sciences, University of Birmingham, Birmingham, United Kingdom

**Keywords:** Robinson Crusoe Island, population genetics, admixture, Chile, Latin America

## Abstract

Studies examining genetic conditions common in Latin America are highly underrepresented in the scientific literature. Understanding of the population structure is limited, particularly Chile, in part due to the lack of available population specific data. An important first-step in elucidating disease mechanisms in Latin America countries is to understand the genetic structure of isolated populations. Robinson Crusoe Island (RCI) is a small land mass off the coast of Chile. The current population of over 900 inhabitants are primarily descended from a small number of founders who colonized the island in the late 1800s. Extensive genealogical records can trace the ancestry of almost the entire population. We perform a comprehensive genetic analysis to investigate the ancestry of the island population, examining ancestral mitochondrial and Y chromosome haplogroups, as well as autosomal admixture. Mitochondrial and Y chromosome haplogroups indicated a substantial European genetic contribution to the current RCI population. Analysis of the mitochondrial haplogroups found in the present-day population revealed that 79.1% of islanders carried European haplogroups, compared to 60.0% of the mainland Chilean controls from Santiago. Both groups showed a substantially lower contribution of indigenous haplogroups than expected. Analysis of the Y chromosome haplogroups also showed predominantly European haplogroups detected in 92.3% of male islanders and 86.7% of mainland Chilean controls. Using the near-complete genealogical data collected from the RCI population, we successfully inferred the ancestral haplogroups of 16/23 founder individuals, revealing genetic ancestry from Northern and Southern Europe. As mitochondrial and Y investigations only provide information for direct maternal and paternal lineages, we expanded this to investigate genetic admixture using the autosomes. Admixture analysis identified substantial indigenous genetic admixture in the RCI population (46.9%), higher than that found in the Santiago mainland Chilean controls (43.4%), but lower than a more representative Chilean population (Chile_GRU) (49.1%). Our study revealed the Robinson Crusoe Island population show a substantial genetic contribution for indigenous Chileans, similar to the level reported in mainland Chileans. However, direct maternal and paternal haplogroup analysis revealed strong European genetic contributions consistent with the history of the Island.

## Introduction

Isolated populations can provide unique insights into human history and patterns of human migration. The underlying genetic structure of a population provides an important first step to elucidating the genetic basis of conditions common to those isolated populations stemming from a founder effect or population bottle neck. For example, the DECODE project revealed variants in the *ASGR1* gene that are associated with a reduced risk of coronary heart disease in the well-studied Icelandic population (Nioi et al., 2016). These findings in an isolated population lead to the successful development of a new treatment for cardiac disorders (Janiszewski et al., 2019). Similarly, a genome-wide association study in the Sardinian population identified novel loci involved in β-thalassemia (Danjou et al., 2015).

Studies into genetic conditions common to South American nations tend to be underrepresented in the literature which is historically European-centric (Carvajal-Carmona et al., 2000, 2003; Bedoya et al., 2006; Criollo-Rayo et al., 2018). Recently Lorenzo Bermejo et al. (2017) showed that the percentage of indigenous ancestry in modern Chileans is correlated with an increased risk of developing gall bladder cancer (Lorenzo Bermejo et al., 2017). Investigations into population structure form the basis of these studies, and several large recent studies have begun to shed light upon the genetic ancestry of modern South American populations. In particular, the admixture between the indigenous South American populations, the European settlers and the African slaves brought with them (Ruiz-Linares et al., 2014; Adhikari et al., 2017). On average, Chileans show a smaller proportion of African ancestry compared to Colombians. Most recently, the regional indigenous contribution to Chilean ancestry has become better understood. The genetic contribution of indigenous groups (Aymara in the north, and Mapuche in the south) was found to be relative to longitude, geographically correlating with the regions inhabited by these indigenous groups (Chacon-Duque et al., 2018). Similarly, the proportion of indigenous ancestry shows a correlation with socioeconomic status, where people from a lower socioeconomic background are more likely to have a higher proportion or indigenous ancestry, whereas those from a higher socioeconomic background are likely to have a larger European contribution (Lorenzo Bermejo et al., 2017).

These studies have collected data from a range of South American admixed and indigenous populations that provide an exciting resource from which to understand the fine-scale structure of previously unreported and interesting populations. Here, we report the first comprehensive investigation into the population structure and recent admixture of the inhabitants of the Robinson Crusoe Island in Chile.

Robinson Crusoe Island (RCI) is the only permanently inhabited island within the Juan Fernandez Archipelago, located 670 km due east of San Tiago, Chile. Originally named Más a Tierra (Closer to Land) it is the second largest island of the archipelago, after Más Afuera (Farther Out) (Villanueva et al., 2014). The archipelago was first discovered by its namesake, Juan Fernandez, in 1574. The island is thought to be the inspiration behind Daniel Defoe’s 1719 novel Robinson Crusoe. The Scottish sailor Alexander Selkirk spent 4 years and 4 months (1704–1709) marooned in isolation after triggering a mutiny of the ship Cinque Ports (Severin, 2002, p. 5–8). Selkirk refused to continue on a vessel he judged to be unseaworthy and forced the captain, Thomas Stradling, to leave him ashore on the closest island. Selkirk was proven correct when the ship, the Cinque Port sank shortly after. In the 1960s, the island underwent a name change to Robinson Crusoe Island to encourage tourism, and Robinson Crusoe’s Cave remains one of the principal tourist attractions on the island.

Throughout the 17th to 19th centuries, Robinson Crusoe Island was frequently used as a stopping point by buccaneers seeking refuge on their voyage around the Cape Horn, the southernmost tip of Chile, the Wollaston Islands (Woodward, 1969, p. 15–109). Through mutiny, abandonment, or deliberate attempts at colonization, these resulted in many, often short-lived, attempts to inhabit Robinson Crusoe Island. The island was briefly populated from 1760 to 1837, when it functioned as a prison. Conditions were extremely harsh and violence was common, resulting in the island gaining a reputation as hostile and unforgiving (Woodward, 1969, p. 91–175).

The origins of colonization of the modern RCI population occurred in the mid-1800s, although accounts vary considerably between sources. According to Severin (2002), the first ancestor of the current islander population arrived in 1889 (Severin, 2002, p. 29). However, Woodward (1969) reported that this occurred c.1860 when his ship was wrecked and he decided to stay on the island (Woodward, 1969, p. 200). From 1867, Frederick Flindt, a German colonizer rented the island from the Chilean government (Woodward, 1969, p. 204–206). He purchased a ship which he named the Juan Fernandez in 1868 carrying 32 colonists to the island – reported to consist of “21 Chileans, 7 Englishmen, and 4 women” (Woodward, 1969, p. 205) – many descendants of these colonist individuals remain living on the island today.

The island population struggled to stabilize with many departures in the face of harsh conditions – in 1869 there were 130 people inhabiting the island, whereas 4 months later there were only 18 men capable of work and 48 women and children remaining (Woodward, 1969, p. 206). Robinson Crusoe Island finally began to establish a permanent population when it was bought by the Swiss Baron Alfred Von Rodt in 1877 (Woodward, 1969, p. 208), and by the end of that year there were 73 inhabitants. Two years later, there were 141, and by 1885 there were 82 (Woodward, 1969, p. 209).

RCI is both geographically and culturally isolated, with most of the current island population of 926 inhabitants (2017)^1^, being directly related to these original founders and of mixed Chilean, Spanish, Swiss, German and British ancestry (Villanueva et al., 2015a). Villanueva et al. (2014) reported that islanders show a high consanguinity rate of 14.9% and the average inbreeding coefficient (α) is 54. −5 × 10^–4^, indicating that unions between first and second cousins are frequent.

The population of Robinson Crusoe Island have been studied in detail because of an unusually high prevalence of language disorder, estimated to affect one child in three, ten-times the rate found in mainland Chile (Villanueva et al., 2008, 2011; De Barbieri et al., 2018). Genetic studies have identified a risk factor in the gene *NFXL1* conferring an increased risk of language disorder and explaining 17% of the trait variance found on RCI (Villanueva et al., 2015a). As part of the investigation into the genetic cause of language disorder, extensive genealogical data have been collected and near-complete ascertainment of ancestry across the entire island has been achieved (Villanueva et al., 2011, 2014; De Barbieri et al., 2018).

## Materials and Methods

### Ethical Approval

This study was carried out in accordance with the recommendations of the University of Chile Ethics Department for project “Genetic analysis of language impaired individuals from the Robinson Crusoe Island” (Project Number 001-2010). All subjects and/or their parents, where applicable, gave written informed consent, in accordance with the Declaration of Helsinki.

### DNA Extraction and Genotyping

Genomic DNA samples were collected from 163 residents from RCI and 30 Chilean controls (referred to as Chilean) consisting of 15 male university students and 15 female adult controls residing in the Santiago area. DNA extractions were performed using a standard chloroform extraction protocol from EDTA whole blood samples (Villanueva et al., 2015a).

Samples were genotyped with the Affymetrix Axiom GW-LAT 1 array (Affymetrix Inc., Santa Clara, CA, United States)^2^, supplemented with a custom array designed to cover South American-specific variants. Standard quality control procedures were completed within PLINK v1.90b4.4 (Chang et al., 2015), during which any variant with a Minor Allele Frequency (MAF) <1% or a call rate <98% was excluded. Individuals with a genotype rate <97%, unexpected gender or inconsistent genotypes with family members were also excluded. Following quality control, the dataset consisted of 163 islanders and 30 mainland Chilean controls across 1,141,741 SNPs. A total of 29,231 autosomal SNPs were found to overlap between the islander and external control datasets and were taken forward in the ancestry analyses.

### Mitochondrial and Y Chromosome Haplogroup Analysis

To minimize bias and false signal from a high degree of direct relatedness in the RCI group, children (where data was present for at least one parent) (*N* = 81) were excluded from further analysis (remaining sample size, RCI *N* = 86, Chilean *N* = 30). Maternal ancestral haplotypes (RCI *N* = 86, Chilean *N* = 30) were estimated using 180 mitochondrial SNPs using Haplogrep2 (Van Oven, 2015; Weissensteiner et al., 2016). Y chromosomal haplotypes were generated for male individuals (RCI *N* = 39, Chilean *N* = 15) from 270 SNPs contained on the Y chromosome (non-pseudo autosomal region) using Y-fitter (Jostins et al., 2014). Y-fitter uses a maximum likelihood method considering the entire Y chromosome and based on the haplotype tree published by Karafet et al. (2008). The defining SNP used by Y-fitter is indicated in parenthesis after the haplogroup. The ancestral mitochondrial and Y chromosome haplotypes of the original founder families were inferred from individuals with an unbroken maternal or paternal lineage from genealogical data.

### Admixture Analysis

European (CEU) (*N* = 40), Iberian (IBS) (*N* = 14), and African Yoruba (YRI) (*N* = 40) control population datasets obtained from 1000 Genomes^3^. An additional Chilean control population (*N* = 190) containing individuals from across a range of geographic areas were accessed through dbGaP (Evaluation of Ancestry Admixture Among Chileans, phs001385.v1.p1) (Lorenzo Bermejo et al., 2017). From this dataset, 176 Chilean individuals passed quality control, and were grouped into those who self-identified as indigenous (Mapuche, *N* = 32) and those who identified as non-indigenous Spanish-Chilean (Chile_GRU, *N* = 144).

Genetic principal component analyses were calculated using PLINK v1.90b4.4 (Chang et al., 2015) and plotted using the ggplots2 package in R (Wickham, 2016). Admixture analysis (*K* = 3) was performed using the ADMIXTURE software (Alexander et al., 2009) and visualized using ggplots2 (Wickham, 2016).

## Results

### Mitochondrial and Y Haplogroups in the Present-Day Population

To gain an overview of the contributory ancestral populations, we examined the mitochondrial and Y chromosomal haplotypes found in the present-day inhabitants of Robinson Crusoe Island, and compared these to Chilean controls from the mainland (Santiago).

**FIGURE 1:**
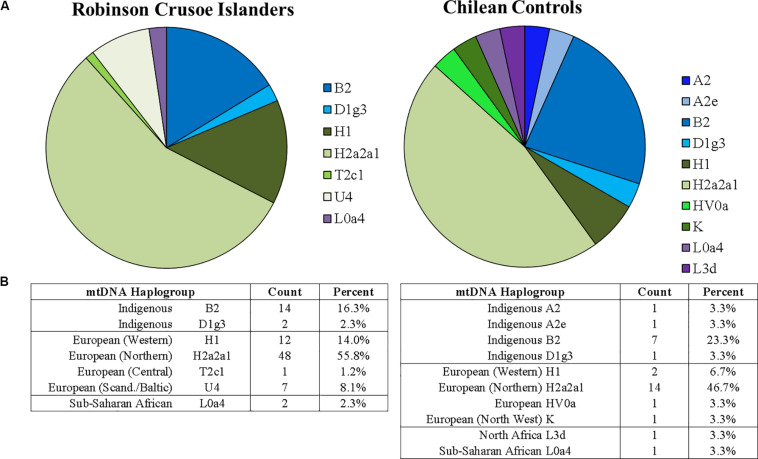
**(A)** Proportions of mitochondrial haplogroups found on Robinson Crusoe Island (*N* = 86) (left) compared to Chilean controls (from Santiago) (*N* = 30) (right). Haplogroup origins are grouped by color: those common to South American indigenous populations (blue), Northern Europe (green) and Africa (purple). **(B)** Counts of mitochondrial haplotypes found in Robinson Crusoe Island and mainland Chilean controls grouped by Indigenous and European origins.

Figure 1A shows the percentage breakdown of maternal haplotypes in islanders (*N* = 86) compared to Chilean controls (*N* = 30). In general, indigenous South American haplogroups were less common in the RCI population than mainland Chilean controls. In particular, mitochondrial haplogroups B2 and D1g3 have been reported to be common to Indigenous America populations (O’Rourke and Raff, 2010; de Saint Pierre et al., 2012; Rishishwar and Jordan, 2017). Both were present in islanders and Chilean controls (shown in blue) but were less frequent in the RCI population compared to Chilean controls, suggesting a higher degree of European ancestry than found in a general Chilean population. Additionally, the common indigenous haplogroup A2, and subgroup A2e were present in one individual each in the Chilean controls but were not observed in the RCI population. This suggests that there may have been a more modest contribution of native South American mitochondrial haplogroups on Robinson Crusoe Island than that seen in a general Chilean population.

The most frequent mitochondrial haplogroups in both populations were the European H2a2a1, which is commonly found in Northern Europeans (Rishishwar and Jordan, 2017), followed by H1, spread across Western Europe, particularly common to Iberia and North Africa (Ottoni et al., 2010). Interestingly, the common Sub-Saharan African origination haplogroup L0a4, which is found at low frequencies in North Africa and Southern Europe (Tishkoff et al., 2007), was found in both the islander and Chilean control populations. Similarly, the North African haplogroup L3d was found in the Chilean Control group (Kujanova et al., 2009) although not seen in the islanders. Collectively, the L subclade is most common in Africa, but has spread to North Africa and is present at low levels throughout Southern Europe^4^.

The rarer European haplogroups HV0a and K were detected only in the Chilean control group. HV0a is found across all of Europe, and K is present across North West Europe, broadly spread across North Africa and the Middle East (Rishishwar and Jordan, 2017). The haplogroups T2c1 and U4 were detected only in the RCI population. T2c1 is found in Central Europe, particularly Italy, extending as far as Iran, Iraq, and the Arab Peninsula (see text footnote 4). U4 is an ancient stone-hunter gatherer haplogroup that is relatively rare in modern populations, but is found in modern Scandinavian and Baltic populations (Malyarchuk et al., 2010).

**FIGURE 2:**
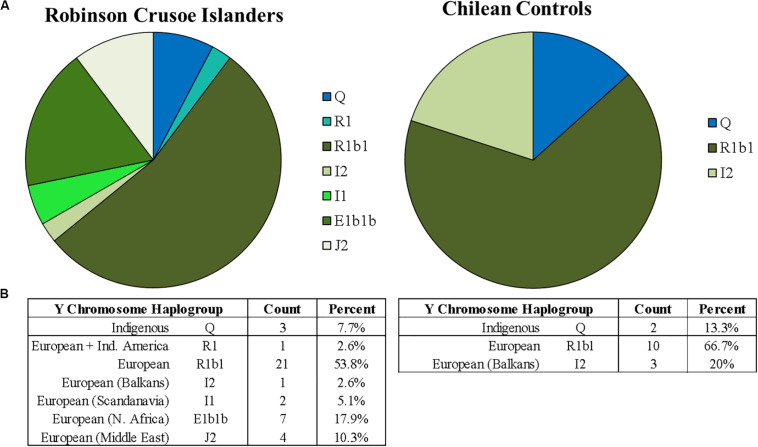
**(A)** Proportions of Y chromosome haplogroups found on Robinson Crusoe Island (*N* = 39) (left) compared to Chilean controls (from Santiago) (*N* = 15) (right). Haplogroups origins are grouped by color: those common to South American indigenous populations (blue), and Northern Europe (green). The R1 haplogroup common to Indigenous Americans thought to be from European admixture is colored in turquoise. **(B)** Counts of Y chromosome haplotypes found in Robinson Crusoe Island and mainland Chilean controls grouped by Indigenous and European origins.

Figure 2A shows percentages of the different Y chromosome haplogroups detected in Robinson Crusoe Islanders (*N* = 37) (left) compared to Santiago Chilean controls (*N* = 15) (right) as reported by Y-Fitter. The common native Indigenous South American Y haplogroup Q (defined by M242) was detected, and was common to both islanders and controls (Bortolini et al., 2003). Interestingly, one Robinson Crusoe Islander was found by Y-Fitter to carry the R1 haplogroup (defined by M173) which is basal to the common European subclades R1a and R1b. R clade haplogroups are common in both North (Malhi et al., 2008) and South American indigenous (Vieira-Machado et al., 2016) populations. They are the second most common Y haplogroup detected in South American males (Malhi et al., 2008) and is considered to be as a result of early European colonization (O’Rourke and Raff, 2010; Vieira-Machado et al., 2016). R1 (M173), however, is a rare haplogroup.

The R1b1 (defined by P297) haplogroup was found in both groups. This is the most common haplogroup in Europe and is widespread across Northern and Southern regions. The less frequent group, I2 (M438), was also present in the RCI and Chilean control groups. I2 is found all across Europe, but is considered a predominantly North European haplogroup, particularly in the Balkans region (see text footnote 4) (Poznik et al., 2016). In contrast to the Chilean controls, several other common European haplogroups (E1b1b, I1, J2, and I2b) were found in the Robinson Crusoe individuals. E1b1b (M215) is frequent in Europe but particularly common in Northern Africa and Southern Europe. I1 (M253) is present in Northern Europe and very common in Scandinavia, with the common European haplogroup J2 (M172) more frequent in the Middle East (Poznik et al., 2016) (see text footnote 4). The rarest haplogroup is I2b (M438), is a minor subclade found in Central Europe (see text footnote 4).

Both mitochondrial and Y chromosome analyses (Figures 1B, 2B) suggest a higher contribution of European ancestry to Robinson Crusoe Island than seen in the Chilean control group. The analysis of mitochondrial data revealed the island population to predominantly carry ancestral haplogroups common to Europe (79%, *N* = 68/86), with some influences from indigenous haplogroups (18%, *N* = 16/86), and common African haplogroups (2.3%, *N* = 2/86). Overall, the Chilean control groups showed a lower proportion of European haplogroups (60%, *N* = 18/30), and a higher proportion of both indigenous (33.2%, *N* = 10/30) and African haplogroups (6.6%, *N* = 30). The Y chromosome ancestral groups told a similar story with the proportion of Indigenous South American haplogroups being substantially lower in the islanders (7.7%, *N* = 3/39) compared to 13.3% (*N* = 2/15) in the Chilean Controls.

### Inference of Founder Mitochondrial and Y Haplogroups

Previous studies, which included genealogical interviews, reported eight founder families were the original island colonizers (Villanueva et al., 2011, 2014, 2015a). Here we use the genetic data to detect distinct founder lineages which can be compared to this historical perception of founding individuals.

**FIGURE 3:**
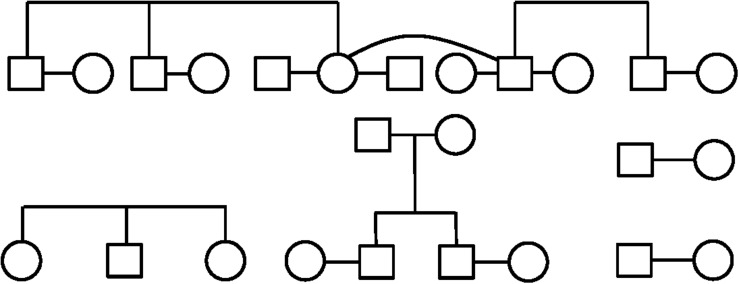
The familial relationships of the founders of Robinson Crusoe Island, who were identified by genealogical analysis, and are directly related to the present day population (Villanueva et al., 2008, 2011, 2014, 2015a). Males are indicated by a square, and females indicated by a circle.

The mitochondrial and Y haplogroups were combined with family structure data to infer the ancestral haplogroups of the founder lineages, through unbroken paternal and maternal lines (Table 1). These analyses indicated the presence of 23 independent first-generation founder individuals as shown in Figure 3. Since these are inferred from the genetics and family structure of the current population, any founding individuals who did not contribute genetically to the current island population will not be detected in these analyses. Mitochondrial haplogroups were successfully inferred in 8 out of 13 founding females, whereas 8 out of 10 founding Y haplogroups were identified.

**TABLE 1:** Inferred mitochondrial and Y chromosome haplogroups of founding individuals, and the number of occurrences of each group.

**Mito. Haplogroup**		**Y Haplogroup**	
H2a2a1	5	E1b1b	2
H1	2	R1b1	4
U4	1	I2	1
Unknown	5	J2	1
		Unknown	2

Interestingly, all of the inferred founder haplogroups that were identified were of European origin, the majority being common European mitochondrial H2a2a1 or R1b1 Y chromosome haplogroups (Table 1). None of the indigenous South American haplogroups found in the current island population, B2 or D1g3 mitochondrial groups or the Q Y chromosome haplogroup (Figures 1, 2), were able to be traced back to founders. In reality, this does not directly exclude the presence of Chilean founders, as Chilean individuals often carry European rather than indigenous haplogroups. In addition, two Y and five mitochondrial founder haplogroups were unable to be determined, as there were no continuous and unbroken maternal or paternal lineages. It is possible that these uncharacterized individuals may have carried indigenous haplogroups that we are unable to detect. However, these findings indicate a substantial proportion of European ancestry to the island founders.

### Admixture on Robinson Crusoe Island

Mitochondrial and Y chromosome haplotype analyses only tell us about the direct maternal and paternal lineages, and the ability to infer ancestry and structure from these data is highly limited. We therefore further explored the population structure of RCI using autosomal markers and principal component analysis (PCA).

**FIGURE 4:**
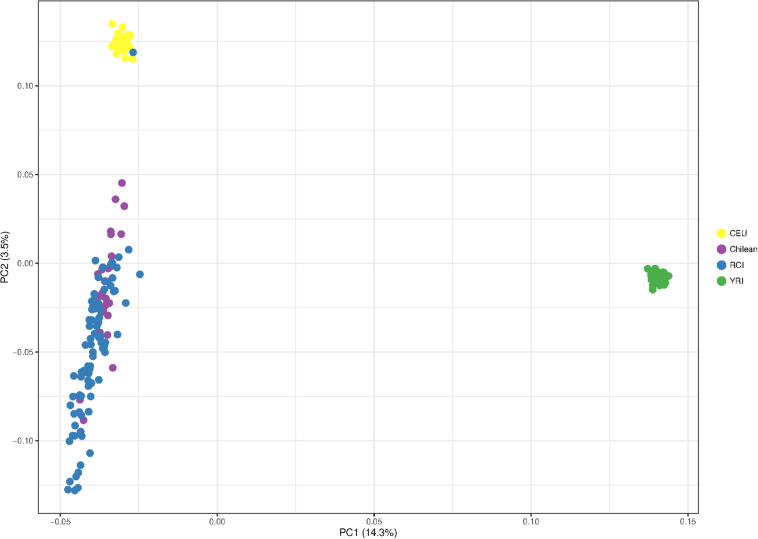
Principal component analysis showing the population structure of Robinson Crusoe Island (RCI) (*N* = 86) (blue) ancestry compared to Chilean controls (from Santiago) (*N* = 30) (purple), European (CEU) (*N* = 40) (yellow) and Yoruba African (YRI) (*N* = 40) (green) 1000 Genomes populations.

Figure 4 shows the principal component analysis of the RCI population (blue) in relation to CEU Europeans (yellow), YRI Yoruba Africans (green), and Chilean controls (Santiago) (purple). The PCA shows a distinct European – African ancestry on the x-axis accounting for 14.3% of the difference between the given populations. The y-axis shows a European – Indigenous component accounting for 3.5% of the difference in the populations. The RC Islanders (with the exception of one typical European individual) overlap substantially with the Chilean controls (Santiago), but appear to have moderately more indigenous ancestry overall.

To investigate this indigenous contribution in more detail, we repeated the PCA including more data from indigenous South American and Iberian Spanish European reference populations.

**FIGURE 5:**
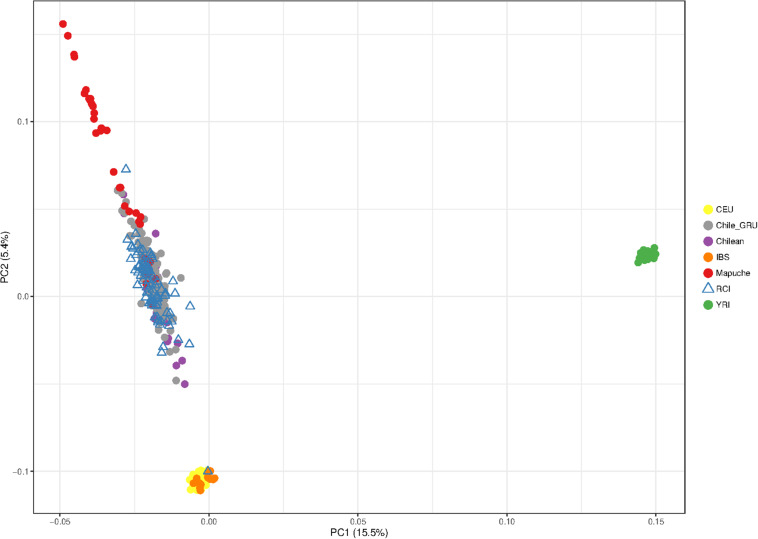
Principal component analysis showing the population structure of Robinson Crusoe Island (RCI) (*N* = 86) (blue triangle) compared to Chilean controls (from Santiago) (purple). Data from the non-indigenous “Evaluation of Ancestry Admixture Among Chileans” (Chile_GRU) (*N* = 144) study (Lorenzo Bermejo et al., 2017) are shown in gray, with individuals from this study who self-identify as of Mapuche indigenous ancestry (Mapuche) (*N* = 32) in red. European (CEU) (*N* = 40) (yellow), Iberian (IBS) (*N* = 14) (orange), and Yoruba African (YRI) (*N* = 40) (green) from 1000 Genomes populations are also shown.

The Chile_GRU individuals represent non-indigenous participants from the “Evaluation of Ancestry Admixture Among Chileans” controls (Lorenzo Bermejo et al., 2017). The indigenous data (Mapuche) were the individuals from this study who self-identified as Mapuche, a tribal group from south-central Chile and southwestern Argentina (Lorenzo Bermejo et al., 2017). The RCI and Chile_GRU (non-indigenous) individuals showed a great deal of overlap with each other, suggestive of similar population structure. Interestingly, both RCI and Chile_GRU (non-indigenous) populations showed a similar spread, with some individuals tending toward a high degree of indigenous ancestry, while others had very little (Figure 5).

The CEU Europeans and IBS Iberian (Spanish and Portuguese) control groups were indistinguishable from each other at this level of resolution (Figure 5).

**FIGURE 6:**
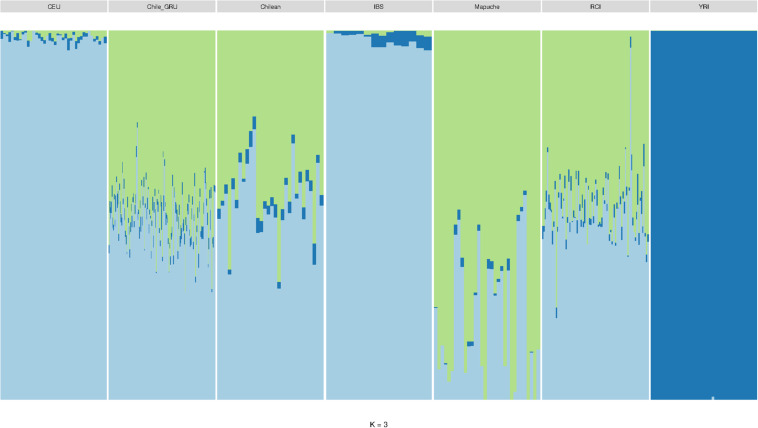
ADMIXTURE bar plot (*K* = 3) showing the estimations of ancestry in Europeans (CEU) (*N* = 40), Chile_GRU (non-indigenous) (*N* = 144), Chilean controls (from Santiago) (*N* = 30), Iberian (IBS) (*N* = 14), Mapuche (*N* = 32), Robinson Crusoe Island (RCI) (*N* = 86), and African Yoruba (YRI) (*N* = 40) populations.

**FIGURE 7:**
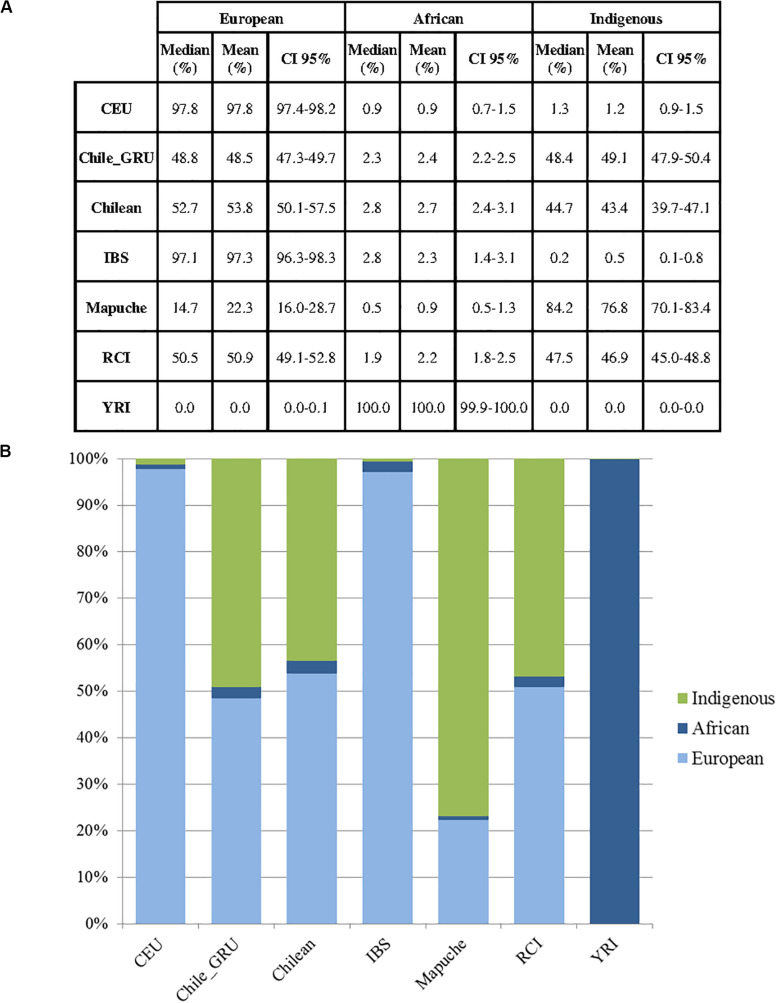
**(A)** Estimates of ancestry summary statistics for populations in Europeans (CEU) (*N* = 40), Chile_GRU (non-indigenous) (*N* = 144), Chilean controls (from Santiago) (*N* = 30), Iberian (IBS) (*N* = 14), Mapuche (*N* = 32), Robinson Crusoe Island (RCI) (*N* = 86) and African Yoruba (YRI) (*N* = 40) populations (*K* = 3). Median, mean and confidence intervals shown. **(B)** Bar plots of estimated ancestry proportions for each population group.

As mitochondrial and Y haplogroup analysis suggested the RC Islanders have a substantial European ancestry, and PCA indicated there was a high degree of individual variability in indigenous ancestry within the RCI group, we performed an admixture analysis (Figure 6). This was performed using ADMIXTURE with an estimated population size (K) as 3 (cross validation estimate is show in Supplementary Figure 1). The RCI group showed a similar admixture pattern to both Chilean controls from Santiago (Chile) and the non-indigenous individuals from the “Evaluation of Ancestry Admixture Among Chileans” study (Chile_GRU). These data therefore indicate there is no substantial difference in genetic ancestry between the present day population of Robinson Crusoe Island and mainland Chile (Figures 6, 7A,B). Robinson Crusoe Islanders showed a mean European ancestry of 50.9% and indigenous ancestry of 46.9%, compared to the Chilean controls of 53.8 and 49.1%, and Chile_GRU (non-indigeous) individuals of 48.5 and 49.1%, respectively. The estimates of admixture per individual can be found in Supplementary Table 1.

## Discussion

### Indigenous Mitochondrial and Y Chromosome Frequencies

Mitochondrial analysis revealed that European haplogroups were more common (79.1%, *N* = 68/86) on Robinson Crusoe Island than Chilean controls (60.0%, *N* = 18/30), although a high degree of European ancestry was evident in both populations. Indigenous South American haplogroups were less common on RCI (18.6%, *N* = 16/86) than in the Chilean control population from Santiago (33.2%, *N* = 10/30). Both these rates of indigenous haplogroups are substantially lower than those reported by previous studies of Chilean populations. Vieira-Machado et al. (2016) reported 88.2% indigenous mitochondrial haplogroups from individuals tested in a number of hospitals in Santiago. Similarly, Rocco et al. (2002) found 84% of mitochondrial haplogroups were indigenous in a mixed Santiago population. There is a striking difference between these studies and the 33.2% indigenous haplogroups found in the Chilean population controls in the current study. This may be due to the demographics of the control population who were students at a University in Santiago. Previous reports suggest that higher proportions of indigenous ancestry are associated with lower socioeconomic status (Lorenzo Bermejo et al., 2017) and that this, in turn, is correlated with educational level in Chile (Contreras, 2001). Haplogroups of an African origin were identified at a low level in both the RCI population (2.3%, *N* = 2/86) and Chilean controls (6.6%, *N* = 2/15), and may represent either Southern European or African admixture.

Analysis of the Y chromosome haplogroups told a more typical story, where European groups were most common, detected in both the majority of islanders (92.3%, *N* = 36/39) and Chilean controls (86.7%, *N* = 13/15). Indigenous haplogroups were present in 7.7% (*N* = 3/39) of RC Islanders and 16.7% (*N* = 2/15) in the Chilean controls. These findings are similar to previous population studies who reported predominantly European haplogroups (8.5% indigenous) (Vieira-Machado et al., 2016) in mixed populations from Santiago (Rocco et al., 2002). The apparent imbalance between maternal and paternal origins has also been observed in other studies of South American populations. Carvajal-Carmona et al. (2000) reported 90% of indigenous mitochondrial haplogroups, compared to only 1% of Y haplogroups, with 94% European and 5% of African origin in the recently founded Columbian population of Antioquia.

The haplogroups reported in this paper were output by Haplogrep2 and Y-Fitter. These methods often assign haplogroups based on the presence or absence of one SNP according to the method and the decision tree they are built upon. To fully resolve and confirm haplogroups it would be necessary to sequence all the defining variants required to assign a subclade.

### Founder Haplogroups

Using extensive, near-complete genealogical records of the island population (Villanueva et al., 2011, 2014), mitochondrial and Y chromosome haplogroups carried by the original founders were inferred from unbroken paternal or maternal lineages in 16 of 23 founding individuals (Table 1 and Figure 3).

The identified founder maternal and paternal haplogroups show an interesting trend. The maternal haplogroups able to be detected through unbroken lineages showed a distinction between Northern European (H2a2a and U4) haplogroups, and the Western European (H1) mitochondrial group. H1 is particularly common on the Iberian Peninsula, although also present at a lower rate across the rest of Europe (Ottoni et al., 2010). The female founders with Western European haplogroups are therefore likely to represent two individuals from Spain, Portugal or Basque region, supporting reports of founders originating from those regions (Woodward, 1969; Villanueva et al., 2008, 2014).

The inferred founder Y haplogroups can be divided into Northern European (R1b1 and I2b) likely representing German, Swiss or British ancestry, and Southern European (E1b1b and J2) indicative of Iberian ancestry. These findings are consistent with male founders being of Northern European (Swiss, German, British), Southern European (Spanish) and Chilean ancestry (Woodward, 1969; Villanueva et al., 2008, 2014, 2015a). However, due to the high rate of European Y haplogroups in South American populations (Carvajal-Carmona et al., 2000; Rocco et al., 2002; Eyheramendy et al., 2015; Vieira-Machado et al., 2016) it is not possible to distinguish Chilean founder males from European.

Interestingly, of the reported eight founding families only six unions were able to be accurately discerned from the genealogical records. This may be because the remaining individual founders recorded by the genealogy were partnered to the offspring of founder couples, and therefore considered a separate family unit. Alternatively, it may be because the recorded founders were ascertained from their relationship to the current island population, therefore any founders without living descendants would have been missed. While either scenario could be plausible, it does not impact this study as only founders who genetically contributed to the current island gene pool are of interest.

We were unable to find any direct evidence to support the reported eight families founding the current population (Villanueva et al., 2011, 2014, 2015a). This is likely a historical perception rather than genetically tractable, and the island genealogy suggests a larger number of individuals contributed to the founding population. This is supported by Woodward’s account of the colonization of the island being established more slowly over the second half of the 19th century than by a single colonization event (Woodward, 1969, p. 200–219). It may also reflect those with a higher social standing as being thought of as the founding families.

Both mitochondrial and Y analyses show a predominant contribution of European ancestry in the Founder individuals. Indigenous mitochondrial haplogroups are more common in both Robinson Crusoe and Chilean populations than indigenous Y haplogroups, likely as a result of the colonization of the Americans by Europeans. It should be noted that carrying a European Y or mitochondrial haplogroup does not exclude a high degree of indigenous ancestry, as European haplogroups (particularly Y) are extremely common in modern South American populations.

### Indigenous Admixture

Principal component and admixture analyses detected a substantial South American native genetic contribution to the current Robinson Crusoe Island population. Ancestry estimates (*K* = 3) showed similar population structure between the current island population, Santiago Chilean controls and the Chile_GRU non-indigenous group. Our findings estimate the genetic contribution from indigenous South Americans to the RC Island population at 46.9%, similar to both Chilean controls (43.4%) and Chile_GRU non-indigenous controls (49.1%). Previous research in outbred Chilean populations identified 40–45% indigenous admixture with European and African estimated at 49–52 and 3% respectively (Eyheramendy et al., 2015; Adhikari et al., 2017; Lorenzo Bermejo et al., 2017; Chacon-Duque et al., 2018). At the autosomal level, we were unable to clearly distinguish between Iberian (IBS) and European (CEU) ancestry at this level of resolution in the RCI population.

Approximately 50% of mainland Chileans perceive themselves as predominantly of European ancestry (Adhikari et al., 2017), and similarly, the Robinson Crusoe Islanders self-identify as European (Villanueva et al., 2014). Our results, similar to studies on mainland Chile (Adhikari et al., 2017; Chacon-Duque et al., 2018), indicate the islanders have a substantial genetic contribution from indigenous admixture.

A previous dental morphology study tested 100 RCI children, using shovel-shaped incisor tooth as a proxy for native ancestry and Carabelli’s cusp as a marker for European ancestry to estimate the ethnicity of the island population (Villanueva et al., 2015b). They found that the islanders predominantly had the European tooth morphology, and estimated indigenous ancestry at 4.3%. This figure is much lower than that detected in the current study in which the indigenous ancestry was estimated at 46.9%. This may be due to dental morphology being a poor marker for ethnicity relative to genetic data.

Both PCA and ADMIXTURE analyses showed a high degree of diversity within each of the Chilean populations including the RCI individuals and control populations (Chilean controls, Chile_GRU, and Mapuche) indicative of a high degree of recent admixture. Within the Mapuche indigenous population, who self-identified as Mapuche, carried a high level of European ancestry. Individually, some Robinson Crusoe islanders showed a predominantly European ancestry, and some a larger proportion of indigenous ancestry (with Mapuche as a proxy for indigenous South American). This reflects the history of how the island was colonized by Europeans, as well as Chileans, who themselves carry a high proportion of European ancestry (Woodward, 1969, p. 205). Low estimates of African ancestry (∼3%) were also detected and these are considered to reflect a low level genetic contribution from the slave trade, and North African admixture into Southern Europe (Adhikari et al., 2017).

There are a number of challenges in the investigation of population structure in Chilean populations and this is compounded in a population such as RCI which includes a recent bottleneck and consanguinity. Individuals in the RCI population were selected on the basis of being as distantly related as possible, however, they are still more related to each other than two mainland individuals. A range of methods are therefore required to delineate ancestral contributions. ADMIXTURE is an allele-based approach which is poor at detecting more subtle structure within a population, but is robust for inter-continental and recent admixture (Lawson et al., 2018). Newer methods such as fineSTRUCTURE (Lawson et al., 2012) and SOURCEFIND (Chacon-Duque et al., 2018) can provide more accurate admixture estimates, but these rely upon availability of data (usually sequencing rather than array) from source populations and relevant control groups.

The main challenge for studying the genetic contribution of native South American populations is accessing appropriate control populations from which to examine population structure. The long and narrow geography of Chile mean there is a large number of distinct indigenous populations, including Aymara in the north and Mapuche in the south. Similarly, the physical barrier of the Andes separating Chile from neighbors Bolivia and Argentina mean this geographical distribution is constrained (Lorenzo Bermejo et al., 2017). Until very recently, relevant indigenous South American populations were extremely limited. Lorenzo Bermejo et al. (2017) showed that by estimating indigenous ancestry using HGDP indigenous population over more appropriate Mapuche and Aymara controls, they underestimated indigenous ancestry by 4.1%. The provision of more closely matched ethnically matched indigenous cohorts, has improved the accuracy of admixture analyses, but the diversity of Latin American indigenous populations are still underrepresented. This highlights the importance of funding open access research into non-European control populations.

The Mapuche dataset was used as a proxy for Indigenous South American populations in this study as this was the only dataset that was publicly available at the time of analysis. By utilizing recently available publicly available data from relevant indigenous populations, we have performed the first complete genetic investigation into the population structure of Robinson Crusoe Island. Despite 200 years in isolation, the current island population revealed a predominantly European genetic background, but with a greater than expected Native American genetic component, and showed a similar structure to that seen in mainland Chile. These findings inform genetic studies of the Robinson Crusoe Island population and that of Chile, moving forward and highlight the importance of using appropriate ethnically matched controls in genetic studies.

## Data Availability Statement

European (CEU), Iberian (IBS), and Yoruba (YRI) control populations can be accessed from 1000 Genomes (www.internationalgenome.org). Chilean population controls (Chile_GRU and Mapuche) (*N* = 190) are available on request through dbGaP (phs001385.v1.p1). The datasets generated and analyzed during this study are not publicly available to preserve anonymity for the Robinson Crusoe Island population. Requests to access the datasets should be directed to DN, diannenewbury@brookes.ac.uk.

## Ethics Statement

This study was carried out in accordance with the recommendations of the University of Chile Ethics Department for project “Genetic analysis of language impaired individuals from the Robinson Crusoe Island” (Project Number 001-2010). All subjects and/or their parents, where applicable, gave written informed consent, in accordance with the Declaration of Helsinki.

## Author Contributions

HM, LC-C, DN, J-BC, and PV conceived and designed the experiments. MF, ZD, PV, and LJ performed the sample collection and genealogical analysis. HM performed the genetic analyses. HM and DN wrote the manuscript. All authors contributed to the article and approved the submitted version.

## Conflict of Interest

The authors declare that the research was conducted in the absence of any commercial or financial relationships that could be construed as a potential conflict of interest.

## Footnotes

1 www.censo2017.cl

2 www.affymetrix.com

3 www.internationalgenome.org

4 www.eupedia.com
