# Supplementary material for: The Genetic Population Structure of Robinson Crusoe Island, Chile
Source: Front Genet. 2020 Jun 26;11:669. doi: 10.3389/fgene.2020.00669 (PMC7333314; doi:10.3389/fgene.2020.00669)
Supplement: Supplementary file 1 [file Data_Sheet_1.docx]

Supplementary Material

# Supplementary Figures


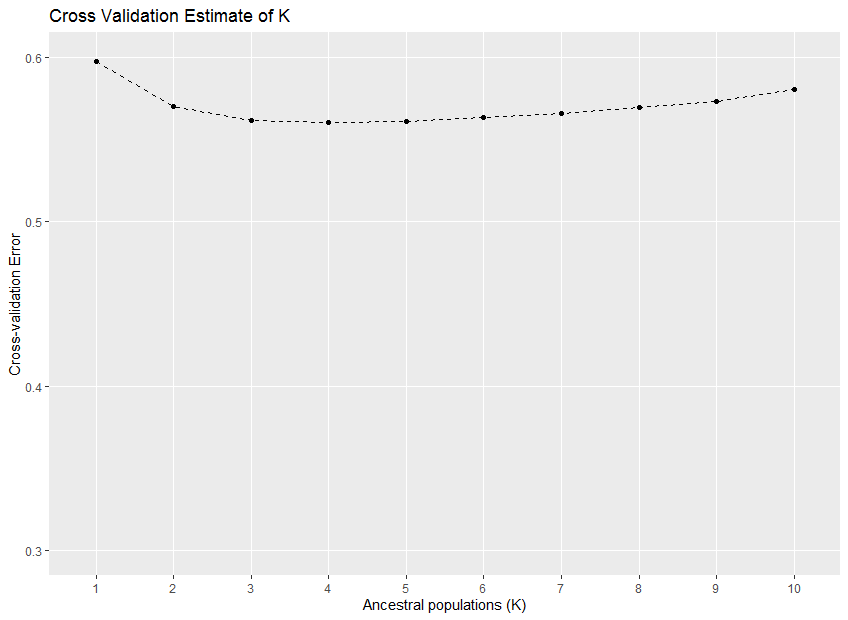


**Supplementary Figure 1.** Plot of ADMIXTURE cross validation estimates for values of population size (K)

# Supplementary Tables

**Supplementary Table 1.** Table showing individual estimates of admixture from each population, calculated using Admixture.
